# Factors for a Successful Laparoscopic Hysterectomy in Very Large Uteri

**DOI:** 10.1155/2017/1637472

**Published:** 2017-09-06

**Authors:** Harald Krentel, Rudy Leon De Wilde

**Affiliations:** ^1^Clinic of Obstetrics and Gynecology, St. Anna Hospital, Herne, Nordrhein-Westfalen, Germany; ^2^Clinic of Gynecology, Obstetrics and Gynecological Oncology, University Hospital for Gynecology, Pius-Hospital Oldenburg, Medical Campus University of Oldenburg, Oldenburg, Niedersachsen, Germany

## Abstract

Minimally invasive hysterectomy is a standard procedure. Different approaches, as laparoscopically assisted vaginal hysterectomy, vaginal hysterectomy, and subtotal and total laparoscopic hysterectomy, have been described and evaluated by various investigations as safe and cost-effective methods. In particular, in comparison to abdominal hysterectomy, the minimally invasive methods have undoubted advantages for the patients. The main reason for a primary abdominal hysterectomy or conversion to abdominal hysterectomy during a minimal invasive approach is the uterine size. We describe our course of action in the retrospective analysis of five cases of total minimal-access hysterectomy, combining the laparoscopic subtotal hysterectomy and the vaginal extirpation of the cervix in uterine myomatosis with a uterine weight of more than 1000 grams, and discuss the factors that limit the use of laparoscopy in the treatment of big uteri.* Trail Registration*. The case report is registered in Research Registry under the UIN researchregistry743.

## 1. Introduction

The disadvantages of abdominal hysterectomy in comparison to the vaginal or laparoscopic approach have been shown in various studies [1]. However in case of very large uteri the abdominal hysterectomy still is the approach of first choice in most cases, although a total minimal-access hysterectomy with all the well-known positive effects for the patients is technically possible. Not only is the uterine weight an important factor of the feasibility of laparoscopic hysterectomy in uteri > 1000 grams, but also other factors such as the flexibility of the anterior abdominal wall, thus the residual intraabdominal volume and, respectively, the ventilation pressure, the correct positioning of the patient, the use of uterine manipulators, thus the uterine mobility, the patients general condition, the encouragement of OR-team, and the surgical experience of the team are highly important. In the following we discuss these extra factors and describe our surgical approach and results referring to 5 cases of minimally invasive hysterectomy in uteri > 1000 grams.

## 2. Method

Retrospective analysis of the outcome of five cases of total minimal-access hysterectomy is realized by the same gynecologic surgeon in uteri of more than 1000 grams.

## 3. Surgical Technique and Results of Five Cases

We realized five total laparoscopic hysterectomies with a median weight of 1422 grams (1035–2100 g.). In all five cases we performed a laparoscopic subtotal hysterectomy with electric morcellation and subsequent vaginal extirpation of the cervix. The median duration of surgery was 194 min (135 min.–237 min.). In all cases we performed an initial diagnostic hysteroscopy to avoid the morcellation of intrauterine malignant pathologies and used an abrasor as uterine manipulator and an electric power morcellator.

In all five cases the premenopausal patients presented with bleeding disorders (hypermenorrhea and dysmenorrhea) and symptoms due to the uterine size such as compression of bladder and bowel and pressure on the sacral plexus (Table 1). The presurgical vaginal and sonographical examination each revealed a large uterus with multiple myomas. Abdominal ultrasound including the kidneys with documentation of a possible presurgical hydronephrosis completed the examination. We disclaimed any radiologic exam.

We performed the supracervical resection of the uterine body after coagulation of the ascending branches of the A. uterina, avoiding contact to the bladder and the ureteral region. The morcellation was performed with a 15 mm reusable electric morcellator applicate through the left inferior incision. Subsequently the cervix was easy to resect by vaginal approach avoiding abdominal contact to the ureter, followed by peritoneal closure and suture of the vaginal cuff. The localization of the optical trocar depended on the cranial uterine extension. We choose an incision in the median line between umbilicus and xiphoid process epigastric area or an alternative first approach in the Palmers Point. In three cases we used 2 auxiliary trocars (5 mm), in two cases 3 auxiliary trocars, and in each case a 5 mm optical system (Table 2). Thus the total length of incision was 15 to 20 mm. The number of auxiliary trocars depended on the uterine mobility. The second and third trocars were placed in the lateral abdominal wall. The fourth trocar was placed in the suprapubic region in order to manipulate the uterine body by application of a twist drill. All trocars used were reusable. The pathologic results revealed leiomyomas in all cases and adenomyosis in case 4. In case 1 the surgery was combined with difficult resection of intraligamentary fibroids and a final diagnostic cystoscopy in order to assure the normal ureteral function. In cases 2 and 3 the surgery was combined with laparoscopic adhesiolysis and adnexal surgery. We had no minor or major complications. All patients recovered rapidly and were very satisfied with the result.

## 4. Discussion

A good surgical result is always based on a correct indication. In case of very large fibroid uteri the evaluation of the possibility of a minimally invasive approach has to consider the uterine size, uterine mobility, the patients clinical history (prior surgeries and adhesion), and general physical conditions. The examination in general anesthesia by the experienced surgeon must be part of the presurgical evaluation. The success of the total minimal-access hysterectomy in uteri of more than 1000 grams also depends on the following factors: surgical experience of the team, instrumental equipment, positioning of the patient, uterine mobilization by a manipulator, and the flexibility of the anterior abdominal wall, the intraabdominal residual volume and ventilation pressure respectively. The duration of the surgery also depends on these factors and not only on the uterine weight. Interestingly we were able to realize the fastest surgery in the largest uterus. De Wilde showed the possible complications depending on the duration of a surgery [2]. Therefore we realized an intermediate mobilization of the patients legs and an occasional change of the positioning after 2 hours to avoid nerve lesions and other complications like compartment syndrome. In different studies a longer operating time in laparoscopic hysterectomy compared to vaginal and abdominal hysterectomy has been shown [3]. Our results also show that the duration does not directly depend on the number of auxiliary trocars, as we realized the fastest surgery with only 2 lateral auxiliary trocars and the uterine manipulator. The mean surgical time of 194 min. had no negative effect on the patients in these five cases. Our patients benefit from the advantages of the minimally invasive approach with excellent esthetic results, less pain, faster recovery, and the prevention of adhesion and related complications such as pain and bowel obstruction [4, 5]. However a certain time limit which is not well defined so far should not be exceeded in order to avoid complications. Thus the expected surgical time could be a contraindication for the laparoscopic approach, especially in the hands of the unexperienced surgeon.

Tchartchian et al. described the combined laparoscopic hysterectomy (LACH) in a case of a uterus with a weight of 2400 grams using the so-called switch-over technique [6], which means a number of six trocars in the anterior abdominal wall. The necessity of the switch-over technique does depend not only on the uterine weight but also on the mobility of the uterus, the uterine shape and the flexibility of the abdominal wall, and thus the residual intraabdominal volume. If the uterine mobilization by the manipulator and the median suprapubic drill is possible, the lateral isthmocervical region with the uterine vascularisation can be visualized by a median optical approach using an angled optical system. Thus additional lateral incisions can be avoided and a maximum of three or four trocars is sufficient, which enhances the minimally invasive effects. In case of very large uteri the residual volume in the intraabdominal space is less than normal, which means that the space we work in is very limited. If the anterior abdominal wall is rigid and it can not be elevated adequately by the normal insufflation pressure of 12–15 mmHg, the switch-over technique with lateral application of the optical system can help to assure a good visualization in a small lateral space. Although the insufflation pressure can be increased temporarily, this has a negative effect on the ventilation pressure and the blood circulation of the peritoneum and thus can increase the risk of postsurgical adhesion.

In all cases we preferred to combine the laparoscopic subtotal hysterectomy with the subsequent vaginal extirpation of the cervix, instead of performing a total laparoscopic hysterectomy. The most important surgical step in laparoscopic hysterectomy is the devascularisation of the uterus. After the coagulation and dissection of the uterine arteries the risk of bleeding is minimal. In case of a total laparoscopic hysterectomy the preparation of the bladder and the paracervical region would be the next step. As the preparation in big uteri is more difficult than normal, the laparoscopic subtotal hysterectomy is easier to perform and can avoid bladder and ureteral complications. Cipullo et al. described a higher rate of major complications in TLH compared to LASH [7]. Morelli et al. showed similar results in comparison to the vaginal hysterectomy depending on the experience of the surgeon [8]. In particular, in large uteri, a higher rate of complications in total laparoscopic hysterectomy can be expected [9]. Otherwise, Sinha et al. concluded that the TLH can be performed by experienced surgeons regardless of the uterine size [10]. In the presented cases, we had no minor or major complications such as bleeding, infection, ureteral, bladder, or bowel lesions. The total minimal-access hysterectomy as a combination of the laparoscopic supracervical hysterectomy and the vaginal resection of the uterine cervix is as safe as both well-known procedures themselves. Tchartchian et al. showed the very low rate of complications and a high rate of satisfied patients after LASH [11]. The resection of the cervix as part of the vaginal hysterectomy is also a safe and routine method. Performing the total minimal-access laparoscopic hysterectomy especially major complications like ureteral lesions can be avoided.

A condition of the total minimal-access hysterectomy is the morcellation of the uterine body with an electric morcellator in order to extract the tissue from the abdominal cavity. Because of potential tissue dissemination within the abdominal cavity, the Food and Drug Administration recently warned against the use of electromechanical power morcellation in hysterectomy and myomectomy [12]. The risk of iatrogenic tumors in the abdominal cavity after morcellation by implants of lost morcellated tissue fragments has been discussed in many publications in the last years. Disseminated uterine tissue fragments can cause symptomatic peritoneal and retroperitoneal myosis, adenomyosis, endometriosis, and endosalpingiosis [13]. Theben et al. described a risk of 0.25% of unexpected malignancies in patients planned for laparoscopic subtotal hysterectomy performing presurgical Pap-smear, transvaginal ultrasound, and abrasion. In 1584 cases, they found two unexpected endometrial malignant lesions and two leiomyosarcomata [14]. Lieng et al. reported a rate of only 0.02% of unintended morcellation of leiomyosarcoma in 4791 LASH [15]. The steering committee on fibroid morcellation of the European Society of Gyneacological Endoscopy (ESGE) concluded that the prevalence of uterine sarcoma in presumed fibroids is 0.14% with a range from 0.49% to 0.014% [16]. Bojahr et al. detected an overall malignancy rate of 0.13%: 0.06% sarcoma and 0.07% of endometrial carcinoma were found in 10731 cases of laparoscopic supracervical hysterectomy [17]. The meticulous removal of all tissue particles after morcellation and a sufficient lavage of the complete intraabdominal space are very important [18]. Uterine size, ultrasound examination, and radiologic methods cannot safely predict uterine malignancy. Presurgical uterine biopsies have a very low sensitivity and therefore cannot help to avoid the morcellation of malignant or premalignant tissues. Hence we realized a diagnostic hysteroscopy in all presented cases to at least minimalize the risk of occult endometrial malignancy. If the laparoscopy reveals suspect uterine tissue, the surgery should be converted to open access and the morcellation should be avoided. These risks have to be discussed as part of the informed consent. Recently several authors described the use of contained in-bag morcellation [19, 20]. This new technique might help to avoid the cell dispersion in intraabdominal morcellation. However the use of bags is limited to a certain uterine size. The alternative laparoscopic-assisted vaginal hysterectomy with extraperitoneal vaginal morcellation or the vaginal removal of the uterine corpus was not feasible in the described cases. A condition for the vaginal approach is the mobilization of the uterine tissue into the pelvis. Due to their size, in the presented cases the uterine bodies were cranial of the pelvis, which made the vaginal mobilization of the cervix impossible. However when the large uterus is easily mobilized, the laparoscopic-assisted vaginal hysterectomy with extraperitoneal morcellation is a safe and cost-effective alternative to the total minimal-access hysterectomy [21] even in nulliparas [22]. If vaginal surgery and laparoscopic in-bag morcellation are not feasible and the patient wishes to avoid laparoscopic intraabdominal morcellation, the abdominal hysterectomy is the only alternative approach in big uteri [23]. Abdominal hysterectomy is associated with longer hospital stay, higher blood loss, and higher analgesic consumption, but a shorter operating time compared to total laparoscopic hysterectomy [24, 25]. In case of abdominal hysterectomy in very large uteri, a longitudinal incision could be necessary, which is associated with an unsatisfactory cosmetic result. When compared to laparoscopic supracervical hysterectomy, the abdominal approach is associated with longer duration of surgery and a higher operative and postoperative complication rate [26]. Compared to vaginal hysterectomy, the laparoscopic approach is less cost-effective [27]. But outpatient laparoscopic hysterectomy has a lower average allowed cost than inpatient open hysterectomy [28]. However the surgical cost in laparoscopic hysterectomy depends on the surgeon volume and thus the individual experience [29]. Additionally the use of expensive single use instruments can be avoided using reusable bipolar coagulation graspers, the monopolar hook or needle, and reusable scissors for tissue dissection. Our patients were very satisfied with the result of the surgery, the advantages of the laparoscopic access, especially the very small incisions, and the fast postoperative recovery. Considering the minimally invasive approaches, vaginal hysterectomy, laparoscopic-assisted vaginal hysterectomy, and total laparoscopic hysterectomy and laparoscopic supracervical hysterectomy, the abdominal hysterectomy can be avoided in almost every case. If the mentioned techniques are not feasible regarding the diagnostic results of presurgical anamnesis, ultrasound, diagnostic hysteroscopy, and diagnostic laparoscopy, the described total minimal-access hysterectomy is a safe and effective alternative in large uteri > 1000 grams.

## 5. Conclusion

In large uteri of more than 1000 grams the total minimal-access hysterectomy is a safe surgical approach with all the advantages of minimally invasive surgery. The feasibility of the method does depend not only on the uterine weight but on a complex variety of factors that must be considered in the process of indication by the experienced surgeon. Thus abdominal hysterectomy can be even avoided in many cases of big uteri.

## Figures and Tables

**Table 1:** Patients data: age, symptoms, past surgical history, duration of hospital stay, and postoperative painkillers use.

	Age	Symptoms	Surgical history	Days of hospitalisation	Use of analgesics
Case 1	46	Hypermenorrhea	None	2	NSAID
Case 2	42	Hypermenorrhea	Laparoscopic appendectomy	3	NSAID
Case 3	39	Hypermenorrhea	Laparoscopic ovarian surgery	2	NSAID
					Metamizole
Case 4	38	Hypermenorrhea, dysmenorrhea	None	3	NSAID
Case 5	42	Hypermenorrhea	None	2	NSAID

**Table 2:** Uterine weight, duration of surgery, and blood loss (^*∗*^presurgical blood transfusion) during the hysterectomy in big uteri.

	Weight (gram)	Time (min)	Hb pre (mmol/l)	Hb post (mmol/l)	Birth	Auxiliary trocars
Case 1	1200	210	8,3	8,5	2x vaginal	3
Case 2	1210	170	8,2	7,6	1x vaginal	3
Case 3	1035	220	9,3	8,2	No birth	2
Case 4	1565	237	2,3	6,5^*∗*^	2x vaginal	2
Case 5	2100	135	7,4	7,3	No birth	2
